# Use of fractals in determining the malignancy degree of lung nodules

**DOI:** 10.3389/fmedt.2024.1362688

**Published:** 2024-03-26

**Authors:** Noel Victor Amador-Legon, Marlen Perez-Diaz

**Affiliations:** Laboratory of Image Processing, Automatic Department, Universidad Central “Marta Abreu” de las Villas, Santa Clara, Cuba

**Keywords:** lung nodule classification, CT, CAD system, fractal dimension, box counting, power spectrum

## Abstract

**Introduction:**

A Computer-Assisted Detection (CAD) System for classification into malignant-benign classes using CT images is proposed.

**Methods:**

Two methods that use the fractal dimension (FD) as a measure of the lung nodule contour irregularities (Box counting and Power spectrum) were implemented. The LIDC-IDRI database was used for this study. Of these, 100 slices belonging to 100 patients were analyzed with both methods.

**Results:**

The performance between both methods was similar with an accuracy higher than 90%. Little overlap was obtained between FD ranges for the different malignancy grades with both methods, being slightly better in Power spectrum. Box counting had one more false positive than Power spectrum.

**Discussion:**

Both methods are able to establish a boundary between the high and low malignancy degree. To further validate these results and enhance the performance of the CAD system, additional studies will be necessary.

## 1 Introduction

Clinical research places lung cancer as one of the types of cancer with the highest morbidity and mortality worldwide, representing about 12.7% of new cases per year and 18.2% of all deaths (1). Lung cancer is defined as a malignant neoplasm, arising as a result of uncontrolled growth of cells in the lung tissue, or the lining of the airways (2). Unfortunately, 80% of detections are in advanced stages. The early detection rate is only 15% (3). If detection occurs in its early stage, when it is called a nodule, survival rates of approximately 75% are achieved (1). According to the Mayo Clinic (4), 60% of people diagnosed with early-stage lung cancer live at least five years after diagnosis. The five-year survival rate for people who are diagnosed with late-stage lung cancer that has spread (metastasized) to other areas of the body is 6%.

In an early-stage lung nodules are approximately round lesions, with a diameter between 5 and 30 mm, which may still be suitable for successful interventions.

**Figure 1:**
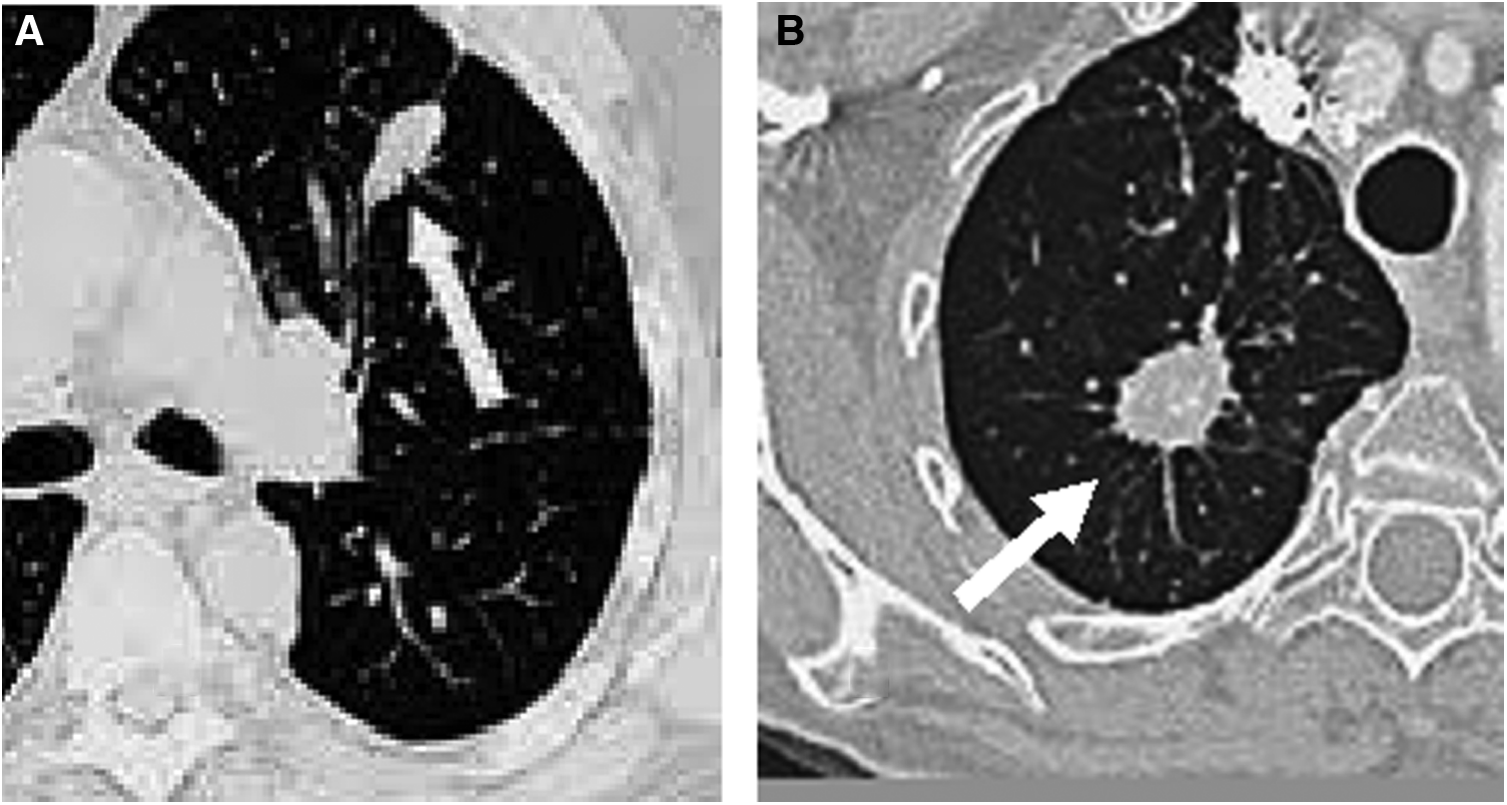
Examples of nodules on CT slices, (**A**) benign and (**B**) malignant.

Medical imaging techniques, such as computed tomography (CT), have been developed for the non-invasive diagnosis of lung cancer. On CT, nodules with non-solid or partially solid content can be distinguished. Both are more likely to be malignant than solid nodules, which are only 15% malignant when smaller than 1 cm (2). The most modern CT equipment are capable of detecting very small nodules, even smaller than 5 mm (5). Figure 1 shows two examples of nodules on CT slices, one malignant and another benign.

Despite all the advances in CT in terms of resolution, speed and availability, the diagnosis of lung cancer continues to represent a problem worldwide. This is due to the overlap of tissues in the thoracic region and the small size of the nodules, as well as the experience of the visualizing specialists, their degree of exhaustion, or viewing conditions. All this brings with it the phenomenon of false negative detection, or misclassification.

To help with problems like the one described above, CAD systems emerged (2). Most CADs are focused on detection, but not on the classification and characterization of lesions. Some that do, take advantage of the morphological and surface characteristics of the lesions to measure their degree of malignancy. Normally, the malignancy degree of a lung nodule is established based on criteria derived from an invasive method for the patient, which is a biopsy.

On the other hand, it has been discovered that most biological structures can be described by scaling analysis (6), which makes fractal geometry a powerful tool for the analysis of biological structures. Its concept does not have both geometric and statistical rigors. Its condition is defined as follows: a statistical property of each small part of an object is not significantly different from the same statistical property measured on the entire object (7).

The term fractal was established by Benoit Mandelbrot in the 80s of the last century (7). Fractals are geometric objects, whose basic structure, fragmented or irregular in appearance, is repeated at different scales. The Hausdorff dimension is a measure of fractal dimension (FD), which was first introduced in 1918 (8). Several methods have been developed to calculate it, which follow the same premise: measure a characteristic at different length scales, plot the points and fit a least squares regression line. The slope of the line will be an estimate of the object FD. Some of the methods that use this principle are: Box counting, Prism counting, Variance method, Power spectrum, among others (10).

A common aspect that all lung nodules have is the alteration of lung morphology (10). These morphological abnormalities can be observed on CT radiological images. It has been appreciated that the large morphological changes caused by tumor growth have an impact on its FD.

This research focuses on tumor shape. Tumors present geometric properties of self-similarity, due to the existence of anomalous roughness in their contour (12). In mathematics, self-similarity, is the property of an object in which the whole is exactly or approximately similar to a part of itself, for example, when the whole has the same shape as one or more of its parts. And this is exactly what a tumor does when it grows. Cells self-replicate and what was once a whole becomes a part.

The shape of its edges has been associated with its malignancy degree (2). Well-defined smooth edges are mostly associated with benign nodules. On the other hand, nodules with spiculated, irregular or lobulated margins are more frequently malignant (1, 12). The spiculated present the most significant margin of malignancy, with a predictive value close to 90%, which has translated into a greater FD (12). This correlation between malignancy and FD has been quantified in studies, giving results of sensitivity, specificity and accuracy for detection of 60%, 76% and 59% respectively (12).

In relation to what has been explained, the objective of this work has been: Develop an automated system in Matlab, based on fractal analysis, capable of classifying the malignancy degree of the detected lung nodules with good sensitivity and specificity.

## 2 Methodology

### 2.1 System overview

The CT slices to be analyzed are subjected to a segmentation stage, to separate the nodule from the rest of the image. In this stage each pixel, according to its luminance level, was filtered to differentiate the nodule from the image background. For this, the Yanni-Horne thresholding method (13) was used. Subsequently, with the object segmented, a manual cutout of the nodule was carried out and the outline of the lesion was extracted, to avoid interference from the rest of the structure of the region in the calculation of the FD. For this the Sobel operator was used. After carrying out these procedures, the FD of the nodules was calculated, using the Box Counting methods and the Power spectrum (14). These methods were chosen for their mathematical simplicity, speed of calculation and ease of programming. The theoretical basis for the procedures are described by Equations (1–10). The programming codes used are publicly available.

#### 2.1.1 Thresholding using the Yanni-Horne method

The technique is based on comparing the image intensity values with a threshold. If the intensity value of a pixel exceeds the threshold value, then the pixel belongs to the object, otherwise the pixel belongs to the background (13). The output image is a binary image, in which those pixels whose value is 1 belong to the object and the pixels whose value is zero belong to the background (15).

The selection of the threshold value by Yanni-Horne was generated from the histogram of the image. The midpoint between the two peaks was initialized:

(1) Gmid=(Gmax+Gmin)/2

Where Gmax is the highest point other than 0, of the entire gray scale and Gmin is the lowest. Thus Gmax−Gmin became the domain of all values other than 0 of the histogram to be analyzed. This point was updated using the average of the peaks to the right and left of Gmid.

(2) $$\mathrm{Gmi}d^{\prime }=(\mathrm{Gpeak}1\;+\;\mathrm{Gpeak}2)/2$$

From this it was obtained that the optimal way to calculate the threshold, was the following:

(3) $$\mathrm{Threshold}\;=\;(\mathrm{Gmax}-\mathrm{Gmin})\overset{\mathrm{Gmi}d^{\prime }}{\underset{g\;\;=\;\;\mathrm{Gmin}}{\sum }}p(g)$$

Where g is the gray scale value, and *p*(*g*) is the probability distribution function, which gives the probability of occurrence of each gray level.

#### 2.1.2 Nodule contour extraction

The Sobel operator was worked with 3 × 3 pixel masks. The masks were designed to detect the maximum at the edges. These were applied vertically and horizontally by convolution with the chosen image of the nodule (matrix ***A***). They were called Gx and Gy when combined. From them it was possible to calculate the absolute magnitude of the gradient at each point and the orientation of said gradient as (13):

(4) $$\mathrm{Gx}\;=\;\begin{array}{ccc} -1 & 0 & 1\\ -2 & 0 & 2\\ -1 & 0 & 1 \end{array}\;\;\times \;A\;\;\;\;\;\;\;\;\;\mathrm{Gy}\;=\;\begin{array}{ccc} 1 & 2 & 1\\ 0 & 0 & 0\\ -1 & -2 & -1 \end{array}\;\;\times \;A$$

The gradient was calculated as:

(5) $$|G|\;=\;\sqrt{Gx^{2}\;+\;Gy^{2}}$$

The edge orientation angle was calculated as:

(6) $$\alpha \;=\;\mathrm{ta}n^{-1}(\mathrm{Gy}/\mathrm{Gx})$$

#### 2.1.3 Calculation of FD by box counting

Each image A was covered with boxes of dimension “*r*” on each side, resulting in a total of *K(r)* boxes, which contained at least 1 pixel of the image. Since A is an image of dimension MxN, the initial value of “*r*” was the smallest of these dimensions. Iteratively, “*r*” was reduced by half, until the distance between two adjacent pixels was reached (14). The log[*K(r)*] value on the “*y*” axis was plotted against the log(*1/r*) value on the “*x*” axis, with *K* being the number of boxes covering the pattern, and “*1/r*” the scale factor, or reciprocal of the size of the boxes. The slope of the line corresponded to the FD and was defined as the amount of change on the “*y*” axis divided by the amount of change on the “*x*” axis, as illustrated in the following equation.

(7) FD=log(K(r))/log(1/r)

**Figure 2:**
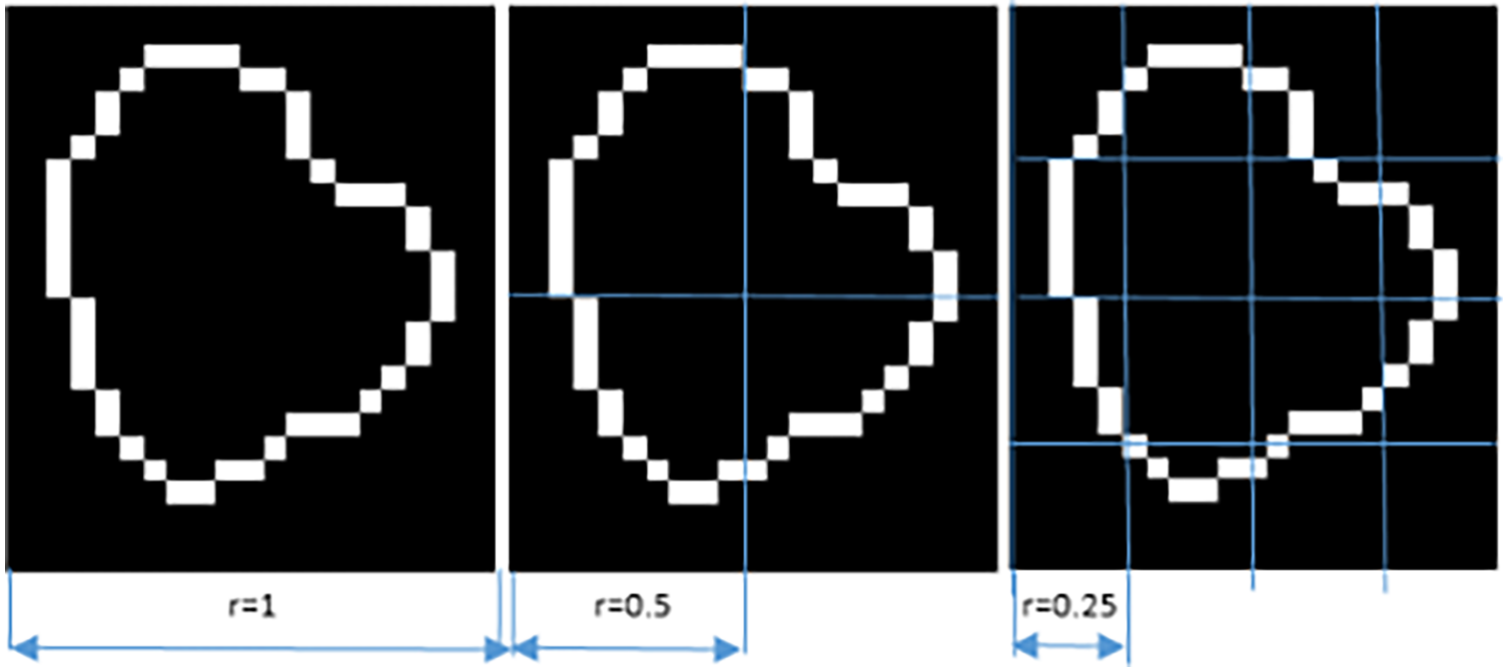
Representation of box counting.

As an example, if a lesion is 5 mm in diameter (approximately 10 pixels) and the maximum division into boxes is up to a distance of 1 pixel, then it is divided up to a maximum of 10 boxes. In order to indicate how the iteration is carried out for the division into boxes, Figure 2 is presented, in three of the N steps to be carried out until the minimum distance of a pixel is reached.

The FD vary from 1 to 2 for this type of analysis. This is a range between a straight line (FD = 1) and a very wavy line (FD = 2), which completely fills a two-dimensional plane. A steeper slope meant that the object was more “fractal”, meaning that it became more complex as the size of *r* decreased. A lower value implied a flatter slope, which meant that the object was closer to a straight line, meaning it was less “fractalized” and therefore its level of detail did not grow as quickly as the magnification increased (9, 14).

#### 2.1.4 Calculation of FD by power spectrum

To convert a contour into a feature suitable for applying the fast Fourier transform (FFT), it is necessary to display it as a function. The display was done through a vector originating from an arbitrary centroid of the contour, which ended in the contour itself (14). The centroid coordinates were calculated in an MxN matrix as follows:

(8) Xctr=1+N/2

(9) Yctr=1+M/2

The vector swept 360 degrees in 1-degree increments, and the magnitude values of the vector were the distribution of the function. Thus, by recording the angle and magnitude values, the function was formed. The calculation of the FD using the power spectrum was carried out through the FFT. The squared logarithm of the magnitude was plotted against the logarithm of the frequency, and fitted to a straight line. Through this linear fit, the slope (ß) was obtained, which has a direct relationship with the FD (14). The ß was related to the FD using the following equation:

(10) FD=(4+ß)/2

Both methods were processed in a Laptop: Toshiba Satellite C75D, Memory: 8GB of RAM, CPU: AMD A7410, eight cores at 2.4GHZ, Hard drive: TOSHIBA MQ01ABD100, 1TB, Video card: 1GB AMD R5 Graphics.

**Figure 3:**
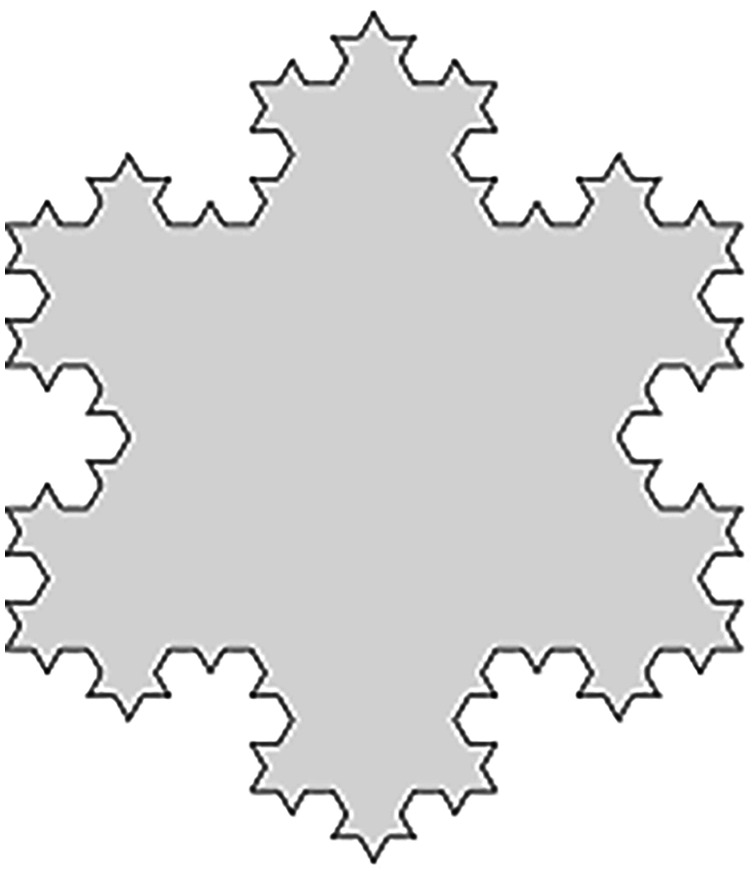
Third-iteration Koch snowflake phantom.

As a reference to prove that both methods work correctly, both were applied on an object of known FD, the Third-iteration Koch Snowflake phantom, simulated with Matlab, as if it were a figure inscribed in a hexagon with 20 mm sideways (FD = 1.2618) (16). Figure 3 shows this object.

### 2.2 Description of the data set used to test the CAD system

Lung Image Database Consortium and Image Database Resource Initiative (LIDC-IDRI) (17) was the lung nodule database used. It also contains the annotation of the malignancy degree of each nodule made by 4 expert radiologists, on a 5-point scale, ranging from highly unlikely-1, moderately unlikely-2, indeterminate-3, moderately suspicious-4, to highly suspicious- 5.

Each nodule has between one and four annotations, depending on the number of radiologists who evaluated the case. When there was more than one annotation, the most repeated value was taken for the present analysis. In this work, the CT scans of 100 patients were selected. Cases 0001–0101 were chosen because they are representative of the 5 degrees of malignancy. For these cases, the slice where the radiologists say that a nodule is best seen in each CT was used. Table 1 shows the distribution of the 100 cases with the malignancy degree noted.

**Table 1:** Malignancy degree.

Malignancy degree	In 100 CT (1 nodule per patient)
1	8
2	26
3	46
4	10
5	10
Total	100

The “Pylidc” graphical interface was used, taken freely from the collaborative development platform Git-Hub (18). Pylidc was developed for using this database, which shows the location of the nodules and allows all slices to be reviewed. It also indicates the slice with the best visibility of each nodule and the annotations made by the expert radiologists about malignancy, texture and internal structure.

### 2.3 Analysis of results

It was necessary to identify the meaning of each calculated FD value. For this purpose, the database annotation (grade of malignancy) was used. Once this was done, the range of FD values obtained for all nodules analyzed by the two methods was calculated, as well as their descriptive statistics: mean and standard deviation. The Pearson correlation between FD and their respective grades of scored malignancy was also calculated for each method. To evaluate the performance of the system, the accuracy, sensitivity and specificity indices were used, following Equations (11–13) (19). They were calculated with respect to the DB annotation, considering 1, 2 and 3 as low degree of malignancy, in other words probably benign, and 4 and 5 high degree of malignancy. The true positives (TP) were nodules identified as positive by the system and were consistent with the DB annotation. The true negatives (TN) were nodules that the system identifies as negative (benign) and that also match the database record. False positive (FP) and False negative (FN) were the classification errors regarding the annotation of the DB.

(11) $$\mathrm{Sens}\;=\;\frac{\mathrm{TP}}{\mathrm{TP}\;+\;\mathrm{FN}}$$

(12) $$\mathrm{Specif}\;=\;\frac{\mathrm{TN}}{\mathrm{TN}\;+\;\mathrm{FP}}$$

(13) $$\mathrm{Acc}\;=\;\frac{\mathrm{TP}\;+\;\mathrm{TN}}{\mathrm{TP}\;+\;\mathrm{TN}\;+\;\mathrm{FP}\;+\;\mathrm{FN}}$$

## 3 Results

The percentage error of each method calculated for the known FD digital phantom was +1.24% for Box counting and −1.59% for Power spectrum.

Table 2 shows the average FD and range for each method for the DB analyzed.

**Table 2:** FD average by malignancy degree in 100 slices (1 nodule per slice).

Malignancy degree	Average FD Power spectrum	Average FD Box counting
1	1.070 ± 0.032	1.095 ± 0.046
2	1.085 ± 0.045	1.125 ± 0.035
3	1.135 ± 0.015	1.146 ± 0.080
4	1.278 ± 0.040	1.256 ± 0.020
5	1.305 ± 0.035	1.270 ± 0.040

As can be seen, as the malignancy degree grew, so did its FD, by both methods. For nodules of adjacent grades, these differences were not very marked and there was some overlap between the ranges, being slightly better in Power spectrum. The FD values were in a similar range those obtained in (12) and (20) for other diseases.

Both methods show strong Pearson coefficients between the five malignancy degree annotated in the database and the FD values obtained. The Pearson coefficient value for Box Counting was *R* = 0.834, while for Power Spectrum it was *R* = 0.908.

Table 3 shows the correct and incorrect classifications made using each method respect to the DB annotation.

**Table 3:** Malignancy classification.

Method	True positive	True negative	False positive	False negative
Box counting	17	74	6	3
Power spectrum	17	75	5	3

From the classification carried out there was a coincidence in five FPs misclassified by both methods and two FNs. Three of the false positives identified and misclassified by both methods have a single annotation in the DB, that is, they were judged by a single radiologist.

Based on the previous results, Table 4 presents the performance evaluation of the proposed CAD system.

**Table 4:** Results of the CAD system performance (%).

Method	Accuracy (%)	Sensitivity (%)	Specificity (%)
Box counting	91	85	92.5
Power spectrum	92	85	93.7

### 3.1 Results of the proposed CAD system

**Figure 4:**
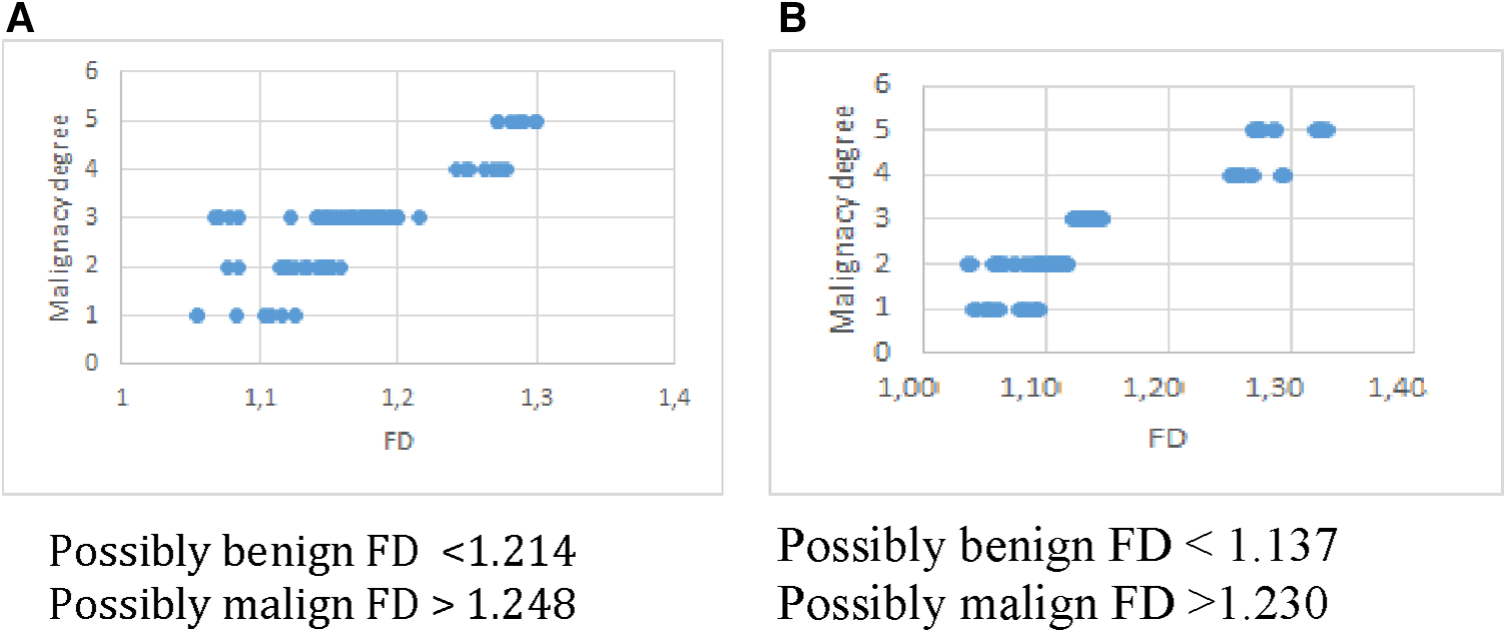
FD range in box counting and power spectrum.

To evaluate the results obtained, it is necessary to apply a classification system. In order to design it, a threshold was established arbitrarily at the upper end of the FD range, corresponding to grade 3 (undetermined malignancy). Thus, a classification system was obtained in two categories, as proposed in (21). For this, the FD ranges obtained from the analysis in Table 2 were used, with the cut-offs recommended by the experts for both methods. The results are as explained below: Probably benign nodules: overlap between the FD ranges for grades 1, 2 and 3. Probably malignant nodules: overlap between the FD ranges greater than the upper value of grade 3, grades 4 and 5. Figure 4 shows this result for Box Counting and Power Spectrum. Misclassified values were excluded from this representation.

**Figure 5:**
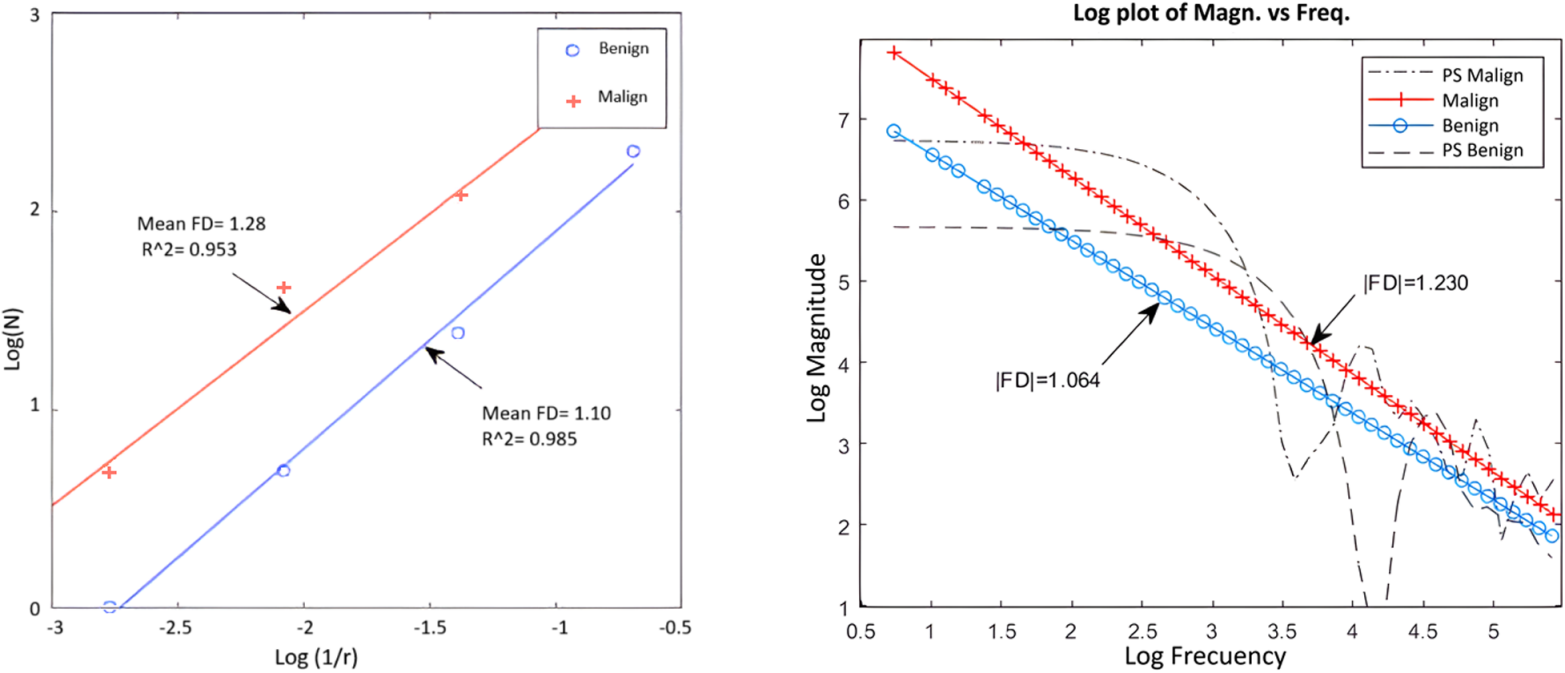
Example of application of box counting (left) and power spectrum (right) for a benign and a malignant nodule.

For two nodules, one benign and one malignant, the calculation of the fractal dimension by both methods has been represented in Figure 5. There is good correspondence between the results of both methods for the same nodules, regarding the class where each one is classified according to the FD value.

As can be seen in general, the Power spectrum had slightly better results in terms of specificity and accuracy, as well as the separation between classes. Although Box counting had one more false positive, in essence both methods have similar performance and showed a high correspondence in results for the DB used.

## 4 Discussion

In the diagnosis of lung nodules from imaging, subjective criteria are usually used to determine the malignancy of nodules (21), as well as invasive biopsies to verify it. In this research, mathematics methods were applied to characterize the nodules, from a quantitative and repeatable approach.

In this work, only the contour of the lesions was focused, leaving aside the internal structure. For this, the contour was successfully extracted using the Sobel operator, that was independent of the internal structure. Studies such as (10, 21) show the use this same approach to evaluate progress in cancer evolution time and response to treatments, with satisfactory results. Studies as (12, 20, 22) report FD values in the same range as that reported in the present work, for the analysis of other pathologies. In our opinion, it is good that the values are not highly dependent on the pathology, as it indicates the potential of using this method in different scenarios, with good generalization power. However, the dependence of the methods on the spatial resolution of the scanner to be used, as well as the degree of precision with which the segmentation method reproduces the contour, must be studied in depth. This last aspect, in our opinion, should be optimized, based on a comparative study of various segmentation methods.

In (23) both the contour approach and that of the entire nodular region were used to characterize the stage in which the tumor was located, comparing it with the local roughness coefficients. Although their results were satisfactory, they were not entirely conclusive. Studies that are based on fractal analysis for classification have limitations, mainly based on the resolution and size of the lesions. To mitigate this problem, artificial intelligence techniques are currently used (24). In the context of this work, where methods are applied to generate a CAD system without using training/validation/testing stages, it is still pending as future work to test the system against a data of different origin, where elements such as noise and spatial resolution are different, to study how image quality influences classification results. Likewise, it will be necessary to study whether the result depends on the nodule segmentation method, testing others.

It is interesting to discuss the boundary found for both methods, where no FD values were found for the DB used. What would happen to a nodule that obtained a FD value between 1,214 and 1,248 for Box Counting or between 1,137 and 1,230 for Power Spectrum? As the number of 100 nodules used to test the methods is considered limited, it is not possible to ensure that obtaining a value in that range is not possible, or on the contrary, that if another database of similar spatial resolution is taken and processed exactly according to the scheme proposed in this work, the limits would not remain reproducible. In this case, the most conservative response is to consider the lesion with a FD greater than 1.214 in Box counting and 1.137 in Power spectrum suspicious for malignancy.

The computational cost with the hardware used was very low, the segmentation and contour extraction took 9 s per image and the FD calculation took 1 s per image with Box Counting and 6 s per image with Power Spectrum, so the CAD proposed is valued as computationally efficient.

The fractal characteristics of the lesions are not the only indicators of the possible malignancy of a nodule. Due to this, studies based on Machine Learning (ML) for the combination of FD with other characteristics are currently more frequent. Studies such as (25) which combine fractal analysis with ML, obtain similar results to those of this research.

The present work is the first part of a more complete system that will include FD as one more radiomic feature from which a machine learning classifier will be implemented.

## 5 Conclusions

It is concluded that our proposed CAD successfully recognize benign and malignant tumors in most of the cases that have been used from a database. Fractal dimensions reflect the characteristics of the lung nodule edges based on the contours irregularities.

## Data Availability

Publicly available datasets were analyzed in this study. This data can be found here: K. Clark, B. Vendt, K. Smith, J. Freymann, J. Kirby, P. Koppel, et al. The Cancer Imaging Archive (TCIA): Maintaining and Operating a Public Information Repository2013. doi: 10.1007/s10278-013-9622-7.
