# Supplementary material for: Use of fractals in determining the malignancy degree of lung nodules
Source: Front Med Technol. 2024 Mar 26;6:1362688. doi: 10.3389/fmedt.2024.1362688 (PMC11002126; doi:10.3389/fmedt.2024.1362688)
Supplement: Supplementary file 1 [file Datasheet1.pdf]

General

```
Y= dicomread('P04-165-N6.dcm'); % image name
I=imadjust(Y,[],[]);

nbins = 256;
k=1;
strel1 = 100;
strel2 = 200;
[M, N] = size(I);

ImClas = class(I);
switch ImClas
    case 'double'
        L = 1;
    case 'uint8'
        L = 255;
    case 'uint16'
        L = 2^16;
    case 'int16'
        L = 2^16;
end

%% Yanni (1994) Iterative Global Thresholding Method.
[Yann_full_image, thrYann] = GlobalBiClasThr_Yanni94(I, k);
% Yanni_full_image = launder(Yanni_full_image, strel1, strel2);
figure(35);
imshow(Yann_full_image, 'InitialMagnification', 'fit'); axis off;
set(35, 'Toolbar', 'none', 'position', [500 -25 370 370])
title({'\bfYanni`s Global Threshold\rm',...
    ['Thresh. = ', num2str(thrYann),', k = ', num2str(k)]});
drawnow

%% Recorte manual
Cropdim = imcrop(Yann_full_image)




[s,a,av,aci] = fdsurfft(BW1)
FDps = (4+av)/2;

%% Box Counting
[D] = hausDim(BW1);
```

Power Spectrum

```
function [slope, intercept, averslope, averIC] = fdsurfft(im)
    tic
    NUM_DIR = 24;
    NUM_RAD = 30;
    if nargin < 1,
        error('Missed input argument which must be an array!')
    end
    [M N] = size(im);

    xctr = 1 + bitshift(N, -1); % x coordinate of center point
```

```
    yctr = 1 + bitshift(M, -1); % y coordinate of center point
    imMean = mean(im(:));
    fim = fftshift(fft2(double(im) - imMean));

    % power spectrum
    mag = log(fim .* conj(fim)+ 10 ^ (-6));
    sumBrite = zeros(NUM_DIR, NUM_RAD); %accumulation magnitude for each
direction and radius
    nCount = zeros(NUM_DIR, NUM_RAD); %number of magnitude
    radius = zeros(2 * NUM_RAD,1);%accumulation magnitude for all
directions
    radCount = zeros(2 * NUM_RAD,1);% number of magnitude for all
directions

    %Compute phase image and phase histogram
    phaseim = zeros(M,N);
    phase = zeros(180);
    for j = 1:M
        for i = 1:N
            realv = real(fim(j,i));
            imagv = imag(fim(j,i));
            if realv == 0
                value = pi/2;
            else
                value = atan((imagv / realv));
                phaseim(j, i) = value;
                ang = floor(180 * (pi / 2 + value) / pi);
            end
            if ang < 0
                ang = 0;
            end
            if ang > 179
                ang = 179;
            end
            phase(ang + 1) = phase(ang + 1) + 1;
        end
    end
    maxphase = max(phase);
    figure; imshow(phaseim, []);
    title('Phase image');
    figure; plot(phase / maxphase);
    title('Phase histogram (0...2 \pi)');%
    axis off

    %accumulation of magnitude for each direction and radius
    rmax = log(min(M,N)/2);% maximum radius
    for j = 1:M
        if j ~= yctr


for i = 1:N
                if i ~= xctr
                    xval = i - xctr;
                    rho = log(sqrt(y2 + xval * xval));
                    if rho > 0 & rho <= rmax
                        mval = mag(j,i);
```

```
if xval < 0
                        theta = theta + pi;
                    end
                    if theta < 0
                        theta = theta + 2 * pi;
                    end
                    ang = floor(NUM_DIR * theta /(2* pi));
                    if ang > NUM_DIR - 1 | ang < 0
                        ang = NUM_DIR - 1;
                    end
                    k = floor(2*NUM_RAD * rho / rmax);
                    h = floor(k/2);
                    if k > 2 * NUM_RAD - 1
                        h = NUM_RAD - 1;
                        k = 2 * NUM_RAD - 1;
                    end

                    if h >= 5
                        sumBrite(ang+1,h + 1)= sumBrite(ang+1,h+1)+
                         mval;
                        nCount(ang+1,h+1)= nCount(ang+1,h+ 1)+ 1;
                    end
                    if k >= 5
                        radius(k + 1) = radius(k + 1) + mval;
                        radCount(k + 1) = radCount(k + 1) + 1;
                    end
                end
            end
        end
    end
end

%linear regression
for ang = 1:NUM_DIR
    sumx = 0;
    sumy = 0;
    sumx2 = 0;
    sumxy = 0;
    sumn = 0;
    for range = 6:NUM_RAD
        if nCount(ang, range) > 0
            yval = sumBrite(ang, range)/nCount(ang, range);
            xval = (range -1) * rmax / NUM_RAD;
            sumx = sumx + xval;
            sumy = sumy + yval;
            sumx2 = sumx2 + xval * xval;
            sumxy = sumxy + xval * yval;
            sumn = sumn + 1;
        end
    end
  slope(ang) = (sumn * sumxy - sumx * sumy) / (sumn * sumx2 -sumx *
         sumx);
    intercept(ang) = (sumy - slope(ang) * sumx) / sumn;
end
```

```
    %compute average slope over all directions and scales
    sumn = 0;
    for k = 6:(2 * NUM_RAD)
        if radCount(k) > 0
            sumn = sumn + 1;
            yval(sumn) = radius(k) / radCount(k);
            tempr(sumn) = (k -1) * rmax / (2 * NUM_RAD);
        end
    end
    p = polyfit(tempr,yval,1);
    averslope = p(1);
    averIC = p(2);
    fitln = polyval(p,tempr);
    figure; plot(tempr,yval,tempr,fitln,'r-');
    title('Log plot of Magn. vs Freq.');
    ylabel('Log Magnitude');
    xlabel('Log Frequency');
    slope(NUM_DIR + 1) = slope(1);
    intercept(NUM_DIR + 1) = intercept(1);
end
Box Counting
function [ D ] = hausDim( I )
    maxDim = max(size(I));
    newDimSize = 2^ceil(log2(maxDim));
    rowPad = newDimSize - size(I, 1);
    colPad = newDimSize - size(I, 2);
    I = padarray(I, [rowPad, colPad], 'post');
    boxCounts = zeros(1, ceil(log2(maxDim)));
    resolutions = zeros(1, ceil(log2(maxDim)));
    boxSize = size(I, 1);
    boxesPerDim = 1;
    idx = 0;
    while boxSize >= 1
        boxCount = 0;

        for boxRow = 1:boxesPerDim
            for boxCol = 1:boxesPerDim
                minRow = (boxRow - 1) * boxSize + 1;
                maxRow = boxRow * boxSize;
                minCol = (boxCol - 1) * boxSize + 1;
                maxCol = boxCol * boxSize;

                objFound = false;
                for row = minRow:maxRow
                    for col = minCol:maxCol
                        if I(row, col)
                            boxCount = boxCount + 1;
                            objFound = true; % Break from nested loop.
                        end;
                        if objFound
                            break; % Break from nested loop.
                        end;
                    end;
                    if objFound
                        break; % Break from nested loop.
                    end;
                end;
```

```
        end;
    end;

    idx = idx + 1;
    boxCounts(idx) = boxCount;
    resolutions(idx) = 1 / boxSize;
    boxesPerDim = boxesPerDim * 2;
    boxSize = boxSize / 2;


end;
for i=1: length(resolutions)-1
    d(i)=log(boxCounts(i))/log(resolutions(i));
end
D=abs(mean(d));
plot(x,y, '*b')
p=polyfit(x,y,1);
y1 = polyval(p,x,s);
plot(x,1y, 'b')
r=1-goodnessOfFit(y1,y,'MSE');
```
